# ‘These are not luxuries, it is essential for access to life’: Disability related out-of-pocket costs as a driver of economic vulnerability in South Africa

**DOI:** 10.4102/ajod.v6i0.280

**Published:** 2017-05-31

**Authors:** Jill Hanass-Hancock, Siphumelele Nene, Nicola Deghaye, Simmi Pillay

**Affiliations:** 1 Medical Research Council South Africa, South Africa; 2 Health Economics HIV and AIDS Research Division, University of Kwazulu-Natal, South Africa; 3 National Department of Social Development, University of Cape Town, South Africa

## Abstract

**Background:**

With the dawn of the new sustainable development goals, we face not only a world that has seen great successes in alleviating poverty but also a world that has left some groups, such as persons with disabilities, behind. Middle-income countries (MICs) are home to a growing number of persons with disabilities. As these countries strive to achieve the new goals, we have ample opportunity to include persons with disabilities in the emerging poverty alleviation strategies. However, a lack of data and research on the linkages between economic vulnerability and disability in MICs hampers our understanding of the factors increasing economic vulnerability in people with disabilities.

**Methods:**

This article aims to present data related to elements of this vulnerability in one MIC, South Africa. Focusing on out-of-pocket costs, it uses focus group discussions with 73 persons with disabilities and conventional content analysis to describe these costs.

**Results:**

A complex and nuanced picture of disability-driven costs evolved on three different areas: care and support for survival and safety, accessibility of services and participation in community. Costs varied depending on care and support needs, accessibility (physical and financial), availability, and knowledge of services and assistive devices.

**Conclusions:**

The development of poverty alleviation and social protection mechanisms in MICs like South Africa needs to better consider diverse disability-related care and support needs not only to improve access to services such as education and health (National Health Insurance schemes, accessible clinics) but also to increase the effect of disability-specific benefits and employment equity policies.

## Introduction

The UN 2015 report on the Millennium Development Goals (MDGs) states that the MDGs have driven ‘the most successful anti-poverty movement in history’ (United Nations 2015) and brought more than one billion people out of extreme poverty. Nevertheless, some groups such as persons with disabilities have been left behind. Acknowledging this and driven by the commitment to leave no one behind, the new Sustainable Development Goals (SDGs) strive for a ‘world that is just, equitable and inclusive’ without discrimination based on any characteristic including disability (Open Working Group of the General Assembly on Sustainable Development Goals 2015).

Persons with disabilities, who also account for one billion people or 15% of the world’s population (World Health Organisation 2011), are thought to live disproportionally (80%) in low- and middle-income countries (LMICs), which also bear the burden of global poverty (IDS 2010). Amongst these countries, middle-income countries (MICs) are home to three-quarters of the world’s population living in poverty (IDS 2010; World Bank 2015). Hence, LMICs and, in particular, MICs bear the global burden of poverty and disability.

Although low-income countries, generally, have few or no mechanisms to counteract poverty or economic vulnerability, MICs are currently developing mechanisms for social protection. This provides an ideal entry point to include persons with disabilities (International Labour Office & International Disability Alliance 2015). Consequently, mitigating the risk of poverty for persons with disabilities in MICs is not only essential but also well-timed and potentially achievable.

Literature suggests that disability and poverty are interrelated in a vicious cycle: with disability increasing the risk of poverty through a lack of opportunities and access, and poverty increasing the risk of disability through poor access to services (health and education) and safe water, risky environments and work conditions and food insecurity (Banks & Polack 2013; Elwan 1999; Graham, Moodley & Selipsky 2013; Groce et al. 2011; World Health Organisation 2011). Recent research indicates that this link is, however, more complex and nuanced than previously anticipated, particularly in MICs (Graham et al. 2013; Groce et al. 2011; World Health Organisation 2011). These nuances are driven by the multidimensional aspects of poverty and disability-driven economic vulnerability which varies in relation to gender, disability type, environmental access and so on (Mitra, Posarac & Vick 2013). As a result of this complexity, more research is needed to better understand economic vulnerability of diverse groups of persons with disabilities so that appropriate social protection mechanisms can be developed to mitigate disability-related economic vulnerability and through this the risk of poverty in these countries (Banks & Polack 2013; Groce et al. 2011; Palmer et al. 2015; World Health Organisation 2011).

Economic vulnerability can be driven by the costs (or resource changes) incurred by the individual or household as a result of disability. These costs can be divided into direct (additional out-of-pocket costs) and indirect costs (opportunity costs) (Palmer et al. 2015). Opportunity costs are understood as the value of the best alternative use of a resource (UCF, Anova Health Institute & WRHI 2015). It is important to understand both sets of costs faced by persons with disabilities, in order to understand the economic vulnerability of these groups.

In the context of disability research, opportunity cost on the individual level is, generally, understood as the income a person could have earned if they did not have a disability (which may reduce opportunities to earn an income). Opportunity cost on a household level can also relate to a caregiver and will be equal to the income that this person could have earned if their family did not include a member with disability that required additional caregiving. Literature on disability has described these disability-associated opportunity costs in LMICs including South Africa, usually in the form of educational outcomes, employment or days out of role (Eide 2003; Eide & Kamaleri 2009; Eide, Khupe & Mannan 2014; Loeb et al. 2008; Mall et al. 2014; Mitra, Posarac & Vick 2011; Mitra et al. 2013). These studies indicate that persons with disabilities are more likely to be amongst those out of school, have lower educational achievements, have less access to health services, have lower rates of employment and have more days out of role. All of these factors translate into lower personal income (Banks & Polack 2013). In addition, a few studies, in South Africa and in other MICs, have shown that opportunity costs are experienced by other household members, where these household members provide care and support at the expense of engaging in income-generating activities (Banks & Polack 2013; De Koker, De Waal & Vorster 2006; Dyson 2005).

Much less is known about the disability-driven out-of-pocket costs that persons with disabilities and their households experience in MICs (Banks & Polack 2013; Palmer et al. 2015; South African Department of Social Development 2016). Out-of-pocket costs are the additional expenses that an individual or household incurs as a result of disability. They can include the costs incurred to enable persons with disabilities to live (e.g. special food and day-to-day support), access services (such as health and education) and participate in society on an equal basis with others (e.g. costs to access employment and recreation) (Banks & Polack 2013).

Currently, we know very little about these costs as in population-based surveys only a small portion of these costs are prompted (e.g. some health expenditures). It is therefore currently impossible to estimate what costs need to be covered by social protection mechanisms to mitigate the economic vulnerability of this population in countries like South Africa. Hence, it is essential to better describe these disability-related out-of-pocket costs from the perspective of persons with disabilities and their household members.

## Enabling the implementation of the Convention on the Rights of Persons with Disabilities

Understanding these costs is also essential to enable countries like South Africa to implement obligations arising through the Convention on the Rights of Persons with Disabilities ([CRPD]; see principles in Box 1). As a signatory of this Convention, South Africa has made considerable steps to domesticate it into its legal framework. In South Africa, the inclusion and equality of persons with disabilities is promoted through the constitution (Constitutional Assembly 1996; Ngwena 2006; Office of the Deputy President South Africa 1997), the White Paper on the Rights of Persons with Disabilities and the Disability-disaggregated National Development Plan 2030 (South African Department of Social Development 2016).

BOX 1 Convention on the Rights of Persons with Disabilities principles.

**CRPD Article 3, General Principles**

The principles of the present Convention shall be:

- Respect for inherent dignity, individual autonomy including the freedom to make one’s own choices, and independence of persons
- Non-discrimination
- Full and effective participation and inclusion in society
- Respect for difference and acceptance of persons with disabilities as part of human diversity and humanity
- Equality of opportunities
- Accessibility
- Equality between men and women
- Respect for the evolving capacities of children with disabilities and respect for the right of children with disabilities to preserve their identities

*Source*: UN Convention on the Rights of Persons with Disabilities 2008

CRPD, Convention on the Rights of Persons with Disabilities.

South Africa has also developed a diverse system of social protection mechanisms to address inequality and poverty, which includes persons with disabilities through targeted grants (e.g. disability and care dependency grant), tax rebates, a housing subsidy and the Employment Equity Act (1998/2016). In addition, South Africa is currently developing a National Health Insurance (NHI) scheme as part of its plans to achieve Universal Health Coverage (South African Department of Health 2015b) that highlights the prioritisation of addressing the needs of persons with disabilities. In addition, the country has developed a comprehensive Framework and Strategy for Disability and Rehabilitation Services (South African Department of Health 2015a), which aims to provide a diverse set of disability and rehabilitation services.

Using this rights-based framework, the country also has to ensure that households with persons with disabilities do not experience higher opportunity or out-of-pockets costs related to their disability. The services that are provided by the state must enable them to participate on an equal basis with others. This is made difficult because of the limited understanding of the economic needs of persons with disabilities and the costs of disability in South Africa and other MICs. Hence, evidence of these needs and of the costs experienced by households is essential for informing the development of inclusive social protection mechanisms, health care and education that are in line with the new legal obligations and frameworks.

The work presented here was conceived within this context and informed South Africa’s ‘project to accelerate the implementation of the Convention on the Rights of Persons with Disabilities’. This project was part of the first round of countries who were supported by the UN Programme on the Rights of Persons with Disabilities (UNPRPD) to promote the rights of persons with disabilities. South Africa focused in this process on the economic vulnerability of persons with disabilities and their households and needed ‘further understanding of the economic vulnerabilities’ (South African Department of Social Development 2016) of this population.

In order to inform this process, a study was conducted that investigated the economic vulnerability of households with persons with disabilities in South Africa (South African Department of Social Development 2016). As a global first in an MIC, this study examined the disability-driven opportunity and out-of-pocket costs as well as the impact of social protection grants on households with persons with disabilities. The overall study included qualitative and quantitative methods of inquiry and a strong community engagement element and rights-based approach. The article presented here uses the qualitative component and describes the perceptions of persons with disabilities with regard to their disability-related out-of-pocket costs in contemporary South Africa.

## Methods

Based on existing literature (Banks & Polack 2013; Groce et al. 2011; International Labour Office 2009; Mitra et al. 2013), we conceptualise poverty within a multidimensional model (Mitra et al. 2013), which takes account of economic deprivation based on access to health care, education, food and natural resources, as well as income-generating opportunities (such as employment).

Using this understanding, the study developed a guiding framework of economic vulnerability which embedded both direct and indirect costs, potential social protection mechanisms and contextual responses (South African Department of Social Development 2016). A scoping review (Hanass-Hancock 2015) informed the development of this framework.

We included a qualitative component in the study which aimed to describe out-of-pocket costs (related to accessing education, health, employment, housing, transport and care and support) from the perspective of persons with disabilities and their caregivers. Within this investigation, we took cognisance of the diverse experiences of varied groups of persons with disabilities. In order to capture these varied experiences, we applied a participatory study design using focus group discussions (FGDs) to develop a consensus of the typical costs that may be borne by households with persons with disabilities, for different types of disability. The study was conducted in close cooperation with the South African Department of Social Development (DSD) and disability sector (Department of Social Development 2016) and included a four-stage process: (1) consultative inception phase with DSD and disability sector, (2) primary data gathering (survey, two-hour FGD), (3) feedback summary and discussion of results with individual Disabled People’s Organisations (DPOs) and (4) consultative validation phase with the DSD and disability sector.

**FIGURE 1:**
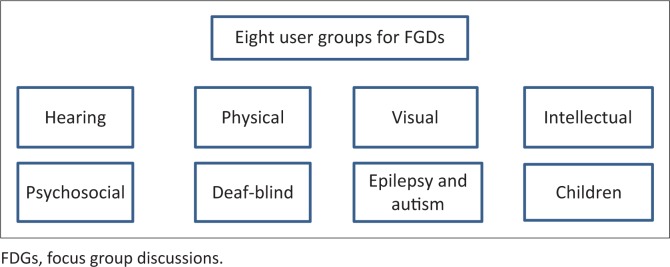
Sampling framework for focus group discussions.

Study participants for the FGD were identified in a two-stage process applying purposive sampling. Firstly, the study approach was discussed with the DSD and disability sector during an inception phase and workshop. At the time of the study, disability programmes fell under the Department of Social Development, which worked in close collaboration with a representative body of DPOs. In the workshop, eight groups were identified for a more in-depth inquiry (Figure 1).

These groups included the representative organisation for persons with each disability type. Participants were recruited from three provinces (Gauteng, KwaZulu-Natal and Western Cape) through the leadership of the representative DPOs. Participants had to belong to one of the identified groups and included persons with disabilities (see Figure 1) who were engaged in community outreach or held leadership positions within the representative DPOs (hence were considered knowledgeable on issues of persons with disabilities). The overarching project also included groups representing children with disabilities, which are not described here. The article uses the terminology for these groups as identified by the disability sector in the inception workshop.

Each FGD, with the exception of the deaf-blind, included 3–14 people with disabilities. FGDs were conducted in the participants’ first language (including sign language) by the researchers themselves and trained research assistants. Persons who are deaf-blind were interviewed on a one-on-one basis to enable effective participation, which because of the nature of the impairment was difficult to enable in a discussion group.

Participation was voluntary and informed consent achieved in two stages. Firstly, potential participants were approached through their respective DPO, and a written information sheet (or alternative, e.g. Braille) explaining the purpose and nature of the study was provided. Secondly, on the day of the FGD, verbal (alternative sign language) information was provided before informed consent was signed and FGD was conducted. Participants were reimbursed for their time and transport.

Overall, 73 adults with disabilities participated in the qualitative part of the study (see Table 1). Each group discussion focused on the specific disability type experienced by the individuals in the group (accessing health care, work and transport) in either a mild or moderate form or a severe form.

**TABLE 1:** Participants in focus group discussions.

Disability group	Number of participants	Province	Gender
Epilepsy and autism, FGD	4 and 2 (2 groups)	Western Cape	3 women, 3 men
Psychosocial disabilities, FGD	6 (2 groups)	Gauteng, Western Cape	5 women, 1 men
Intellectual disabilities, FGD with caregivers	10 (2 groups)	KwaZulu-Natal, Gauteng	8 women, 2 men
Physical disabilities, FGD	20 (2 groups)	Western Cape, KwaZulu-Natal	6 women, 14 men
Hearing disabilities, FGD	14 (2 groups)	KwaZulu-Natal, Gauteng	10 women, 4 men
Visual disabilities, FGD	10 (2 groups)	KwaZulu-Natal, Gauteng	5 women, 5 men
Deaf-blind (individual interviews)	7 (1 group with 2 people and 5 individual interviews)	Western Cape	3 women, 3 men and 1 caregiver

FDG, focus group discussion.

Prior to the discussion group, participants had also completed the cross-sectional survey prompting their personal disability-related costs in the domains of education, work, housing, care and support, health care, transport and employment (South African Department of Social Development 2016). This process stimulated reflection on potential costs, including hidden costs (such as maintenance of assistive devices, or transport costs associated with repeated health care visits), prior to the group discussion. This process helped to address some of the challenges reported in the literature which indicate difficulties in assessing out-of-pocket cost in LMICs because of the lack of knowledge about potential services and assistive devices. During the discussions, participants were encouraged to find a consensus with regard to the cost experienced by a typical individual with their impairment or disability type (in both mild or moderate and severe form) and how this may differ in a rural and urban area. The FGD guide prompted cost of adult education, work, housing, care and support, health care (including assistive devices), transport and employment.

Data from the group discussions were translated into English and transcribed verbatim and analysed by the research team using conventional content analysis identifying themes as they emerged from the data. The quality of the analysis was checked through independent coding by two researchers for each interview.

### Ethical considerations

Ethical approval for the study was provided by the Department of Social Development, and the Ethics Committee of the University of KwaZulu-Natal (HSS/0591/014).

## Results

The study revealed a diverse set of needs and costs that varied not only by degree and type of disability but also depending on the available infrastructure and accessibility of services. What people spent primarily depended on their household income level. Considering the participants’ emphasis on differences between survival and participation, we structure costs in those (1) arising through increased care and support needs (survival), (2) accessing essential services (access) and (3) participating in home and community (participation and dignity). These themes do overlap. However, for the purpose of this article, this structure helps to highlight the participant’s perceptions of the difference between addressing disability-related out-of-pocket costs impacting on survival or access to essential services from those enabling participation on an equal basis to others as defined in the CRPD. It also helped to highlight that some services, such as access to Internet and mobile devices, are for some subgroups (e.g. deaf-blind) not a matter of luxury, but a matter of survival (instead of participation).

### Costs related to increased care, support and assistance (survival)

Spending on care and support was discussed in all groups, as costs were unavoidable and essential for health and survival. These differ by disability type and depend on the specialised skills that are needed to provide care and support for the individual person. In general, persons with severe physical disabilities, dementia, low functioning autism and those who are deaf-blind required specialised and often full-time assistance (which was reported to be very costly), whereas persons with moderate intellectual disabilities or epilepsy needed assistance at particular times or for particular tasks (and the cost was relatively lower). Participants reported that care and support costs mostly take the form of indirect costs, ‘costing’ the time of caregivers (usually a family member). However, if this assistance is provided by another person (not a family member), the direct costs depend on the skills required and time needed.

‘I suppose it will depend on the hours, probably let’s say unskilled person would be three [*thousand rand*] and probably more skilled person would be five or six [*thousand rand, per month*].’ (Mother of person with autism, female, employed)

Participants with high care and support needs who are from lower income households reported that living in an institution is often the only financially feasible way of getting the care needed to stay alive and safe. Care and support needs and the associated costs were closely linked to the available assistive devices and technology. In all discussion groups (except psychosocial disability), participants reported spending on acquiring assistive devices. For some groups such as the deaf-blind, access to communication technology, which is often seen as a luxury in MICs, was seen as essential for communication, staying safe and participation.

‘But in terms of technology deaf-blind people also like a cell phone and iPad and people think, huh you want luxuries. But for blind people these are not luxuries trust me, it essential for access to life. … So it is just a problem technology is extremely important for everything.’ (Person who is deaf-blind, male, employed)

Participants reported not only on the costs of acquiring appropriate assistive devices and technology but also on the cost of their maintenance. In some groups (physical and hearing), these were reported as an important element of disability-driven cost. Those relying on public health care reported waiting long periods of up to three months for maintenance to be completed. Participants elaborated that during this time they would have to live without the device, compromising their health and ability to participate in work and community activities. If they were able to, they would opt to pay for maintenance themselves (or from medical insurance, if available). Participants also reported that persons who cannot afford the continued maintenance, particularly those in rural areas, would opt for other ‘inferior or inappropriate devices’ which could be harmful to their health or cause secondary complications.

‘Yes, you see if you use a wheelchair on these bumpy surfaces of rural roads, wheels get bent easy. So if you are to be taken anywhere around through the wheelchair, expect difficulties ahead. Physically disabled people in rural areas are normally carried away in a wheelbarrow because of the roughness of the land surface. If seriously ill, they may die on it.’ (Person with physical disability, male, peer supporter and temporary employment)

The participants’ descriptions highlight that some of the disability-related care and support costs, including assistance, assistive devices and technology, are essential for their safety and survival. They also shared their perception that the absence of care and support for some disability types such as quadraplegics was directly related to premature death.

‘Quadriplegics die in rural areas. They are left in the bed and they die. Maybe they might last three months.’ (Person with physical disability, female, unemployed)

They also explained that the inability of households to cover disability-related costs from their income threatens survival and increases costs related to access to services and participation in society.

### Costs related to accessing essential services (access)

In all seven FGDs, the personal costs of accessing essential services such as health care, transport and adult education were described. The out-of-pocket costs of accessing health care services were related to increased frequency of medical consultations, specialised travel to health care facilities and additional care and support (while accessing health care services). These were described as increasing with the severity of disability. These costs were high amongst persons who needed more frequent health visits, in particular persons with physical disabilities, intellectual disabilities and epilepsy, and those who are blind or deaf-blind. Reported costs were the lowest amongst those groups where the person with disability could travel to and use the clinic independently (without an assistant) and where there is less frequent need for health care. In addition, stock deficiencies and long queues were reported as causing unnecessary repeated health care visits and its associated costs. To circumvent these problems, in some cases, persons opt to acquire medication and care from the private sector at a higher cost.

‘It is also very time-consuming as public health care facilities are often characterised by long queues and unavoidable delays. In addition, people with epilepsy (especially in rural areas) rely on primary healthcare clinics/facilities for their medication and can be confronted by lack of stock, necessitating a second or even third trip to the clinic. Some people just get told especially in the rural areas that if the clinic or hospital does not have the medication then they must buy it themselves. The person getting a disability grant ends up spending R400–R600 on the medication as they do not want their seizures to increase.’ (Person with epilepsy, male, in learnership programme )

Many groups discussed the use of assistants to allow them to use health care services effectively. Spending on an assistant to support the health care visit was reported as potentially very high. In almost all groups, some participants reported paying for this assistance, even though (in most cases) it is a family member or friend who provides the assistance. Those who do not pay the assistant directly reported providing food, drinks or a gift for the accompanying person. An exception was persons who have psychosocial disability and those who are deaf-blind. Amongst these groups, there was little expectation of ‘donations’ for assistants.

‘People will need an assistant to get to the clinics, which can cost R200 a day …. if you don’t get Dial-a-ride [*subsidised transport*] you have to use private transport up to R500… It would be R20 for the person and R20 for the assistant with Dial-a-ride …’ (Person with physical disabilities, male, unemployed)

In addition, participants reported that patient transport vehicles (provided by the Department of Health) were not necessarily wheelchair-accessible and that there are no other accessible services that regularly service the hospital routes. In these cases, private cars had to be hired or the person was not able to use the health facility. Participants reflected that in rural areas these costs would be even higher because of longer distances to health care facilities.

‘Even hiring a car to take you to hospital costs higher. Even calling for an ambulance can be challenging cause if you stay down the hill where can only be reached by foot, it is difficult. Let me tell you something I haven’t been in a rural area because of such conditions.’ (Person with physical disability, male, unemployed)

Transport to hospital was particularly costly, across most disability types. These costs were reported to be particularly high in emergency situations, when public services were not able to respond and when private sector ambulances had to be called. The cost of private ambulance services applied more to some disability groups (such as those with epilepsy) than others. Considering that some disability groups may need to make use of emergency services more often than persons without disabilities, this may be considered an additional disability-related out-of-pocket cost.

‘I was very sick at home and everyone was still putting me in an ambulance, close to my house on the road and I was still getting seizures, number one, number two and the guy sitting with me inside just said [*name withheld*], relax you will be fine. So afterwards I rang to find out the price of when they came to pick me up and it was about R3000 or something.’ (Person with disability, female, in learnership programme)

Use of health care was most frequent amongst persons with physical disabilities and those with psychosocial disability (where almost all are on chronic medication and where increased use of dentistry is reported). The study revealed that average monthly travel to health care facilities is highest amongst persons with moderate and severe physical disabilities. For other groups, these costs were ‘blurred’ through the use of family members as assistants who provide ‘professional services’ in the absence of affordable support and assistants. For instance, persons who are deaf-blind and those who are deaf emphasised the need for a sign language interpreter for health care consultations. Often, these interpretations are provided by a family member or friend as formal interpretation services are prohibitively expensive (a rate of R2000–2500 per day). If the person with disabilities were to pay for such an assistant, the cost of accessing health care in these groups would be exorbitant.

‘If you hire an interpreter for one day it about R2500 for one day for two are R5000 for every day or per day… Cheaper interpreter means skill is very low, you can get a cheaper interpreter but they have limit to the skill, the information that they give you and information that comes from me will not be to a good quality.’ (Person who is deaf-blind, male, employed)

Three groups of participants, including caregivers and persons with intellectual disabilities, those with autism and those who are deaf-blind, commented on the high cost of adult education. For instance, participants revealed that education centres that accommodate the needs of persons with intellectual disabilities are often far away, hence associated with higher transport costs or costs for accommodation. Similar information was provided by participants in the deaf-blind group and those with low functioning autism.

Participants also raised concerns about the quality of the education that learners with disabilities (more severe disabilities) receive in special schools. These caregivers elaborated that their ‘children’ were ‘just being kept busy’ in these schools and were not prepared for adult life. Participants mentioned that they therefore make use of adult teaching centres for their ‘children’s’ education after the attendance of public schools. Centres such as *Pave It, Cateji* and *I-Can* were mentioned as useful adult education centres that offered training and learnerships for post-secondary schooling. These learnerships are supported through state funding or contributions from employers who support these programmes as part of their social development encouraged through the South African Black Economic Empowerment (BEE) strategies. Again the transport to these scattered facilities was reported as an additional cost. Some facilities tried to overcome this through providing transport, and others encouraged their learners to use subsidised or public transport. However, they experienced multiple challenges with this approach.

‘It [*subsidised transport*] is not reliable, so we as a company have been given three vehicles [*a donation*]… but the company has to provide by employing the driver and cover the petrol and insurance and it is a big cost … and who’s going to cover the cost of running the service?’ (Caregiver of adult with intellectual disability, female, self-employed)

Hence in order to reduce the costs of accessibility, we need to provide better universal design within our mainstream services of health and education, but also adapted designs and assistance particularly in key related services such as transport.

### Costs related to participation in the home and community (participation and dignity)

Participants reported that being able to access home and community depends on the accessibility of these environments. Costs related to housing, transport and assistive devices and technology were discussed. These costs varied depending on the design of environments. For instance, where environments are physically inaccessible (e.g. no ramp and bumpy roads), alternative means of access have to be found. These result either in increased support costs or in the person not being able to access these environments.

‘I would love to just go out and go to the park, but once you have thought about what it cost for your transport, and for your assistant and for your wheelchair …, you rather decide to just stay at home.’ (Person with physical disabilities, female, unemployed)

Similarly, accessible housing was mentioned as a necessity in the groups of persons with physical disabilities. Participants revealed that this group needs adaptations in their houses (wider doors, accessible toilets, kitchens, ramps, etc.), and these can be expensive and are not covered by the state. Again in the absence of these adaptations, care and support needs and costs were higher. Participants reported that for those earning salaries above the income tax threshold, a portion (33%) of the costs for adaptation or support can be reclaimed in the form an income tax rebate. But this (partial) relief is not available to those whose income is below the income tax threshold or who are unemployed.

Some participants explained that some poorer households are able to access state-provided low-cost housing (known as RDP developments). Although these RDP housing developments are meant to provide affordable accommodation to people from poorer households and policies include the provision of accessible housing, participants reported that in these housing estates basic infrastructure such as accessible toilets was still lacking.

‘Yeah, since the government is improving infrastructure like building the RDP houses, some things are not accessible for us as the disabled. For instance those RDP toilets are not accessible for us disabled people especially the steps leading to the toilets.’ (Person with physical disability, male, peer supporter and temporary employment)

Negative attitudes to adjusting these facilities in retrospect, or the absence of personal finance to do so, led to quite undignified situations. In particular, persons with physical disabilities and those ‘living with HIV who are getting worse’ were reported to have difficulties accessing these toilets because of the doors and steps leading to it. One participant further stated that requests for adjustments or modifications were refused, whereas another participant explained a similar situation and how persistent he had to be until an accessible toilet was provided.

‘While I approached the ward councillor regarding this matter and I explained to it as what sort of a toilet to be built for my condition and should be spacious. So if I needed to use the loo I had to stand outside while raining because of the size of the toilet.’ (Person with physical disability, male, peer supporter and temporary employment

Participants highlighted that these issues are worse for people with physical disabilities in rural areas. Non-tarred roads were reported as providing particular challenges to this group of people who ‘battle to walk on this terrain’. Use of wheelchairs on these uneven surfaces results in damage to the wheelchair, leading to more frequent need for maintenance (which was reported as not always promptly provided by health facilities). Participants reported that ‘because of the roughness of the land surface’ in these areas, people use their wheelchairs only at home and use a wheelbarrow outside the home or alternatively they stay indoors most of the time. Hence, activities outside the home were perceived as very costly.

These challenges were exacerbated when public transport was inaccessible, an issue that was raised in most discussion groups and affected all life spheres. Participants with high support needs (and particularly those with physical disabilities or blindness) have to pay their own transport fares and an additional fare for their assistant or an additional fare for their assistive device (wheelchair, guide dog). In some urban areas, participants explained that accessible buses are available, but only at specific time or on limited routes. Although this was acknowledged as a step forward, the scarcity of these accessible buses results in extra costs for the person with a disability. One participant explained that in order to make use of these buses, he had to leave early for an appointment and spend the whole day at his destination until the accessible transport was on its return trip. This would create costs related to time as well as care and support. Within the group of persons with visual impairment, an interesting outlier was reported. Some people in one of the urban settings had access to specialised and subsidised transport. This specific group of people was enjoying cheaper transport than the general population. This transport enabled them to get to work and back, which meant that they were able to work and did not need to spend money on an assistant.

‘I pay R10 from Chatsworth to Umbilo for Dial-a-ride. They fetch me from my house and they stop right outside our building. The driver makes sure that you have got out safely before he drives away. If you need it, he will even assist you to walk all the way into the building. But I am quite independent; I don’t need help to get into the building.’ (Person with visual impairment, female, employed)

For some disability groups, the costs associated with transport overlapped with security issues. For instance, in the groups of people with epilepsy and intellectual disabilities, participants reported that the risk of violence and abuse while using (or waiting for) public transport meant they always had to travel with an assistant or in private cars (if they could afford to buy a car). Further, the cost of adaptation of vehicles had to be borne by the household. For others, the need for a driver or assistant created extra costs.

‘I can also take a taxi but I first used a train and they pickpocketed me. So that is why I am not using a train anymore. We now all take a taxi or a bus.’ (Person with epilepsy, male, in learnership programme)

Transport needs and the need for assistance greatly depended on the available assistive devices and technology. From the discussions, it emerged that access to assistive devices not only decreased the need for (and cost of) assistance and support, but also increased the level of participation and ability to earn a living. For some groups (such as those with severe physical disabilities, those with low functioning autism and those who are deaf-blind), access to assistive devices and communication technology was described as ‘essential for access to life’, hence turning ‘being lonely at home’ into being part of society as an active member that engages via social media with friends and work colleagues.

‘You see that is how deaf-blind works. … On Facebook they were telling me that they [*his peers & colleagues*] need me to support them they were like shouting at me … because I support the Blue Bulls [*a Rugby team*] … they were shouting ‘why are you supporting the Blue Bulls’. We can communicate as the blind-deaf people… you don’t have to feel you are so lonely. That is just an example of what technology can do … So it opens the world it makes the world smaller and brings us together. Unlike the deaf and blind people who are sitting at home no technology at all, they live there and do nothing until they die...’ (Person who is deaf-blind, male, unemployed)

Participants explained that this kind of communication technology requires particular software as well as mobile data packages, a cost that cannot be covered by households who are poor. Hence, access to participation in life for this group is determined by the available technology and income of the household.

The discussion groups also covered the costs of participation in the workplace (for those persons with disabilities who are employed). Across discussion groups, there was agreement that, except for those who were self-employed or for those employed in smaller or informal businesses, the costs of reasonable accommodation were paid by the employer. Some participants reported that the employer’s duty to provide accommodation could lead to employers favouring persons with disabilities who ‘were more independent’ as they want to avoid the ‘extra salary’ for a full-time assistant that may be required by persons with severe disabilities. Others reported on positive changes and provision of employment, despite additional costs.

‘We got people with disabilities into our place it’s mainly because majority are intellectually disabled, it’s attitude, there aren’t many physical costs that are attached to people with intellectual disabilities in the workplace. … I got two people here with intellectual disability working as general workers and if money was not an issue I can do with one but I don’t want to do two people’s jobs.’ (Caregiver of person with intellectual disability, female, employed)

The group of persons with psychosocial disability (mental health) indicated that few persons with this disability type disclose their condition, for fear of losing their job. These groups also explained that accommodation for persons with ‘psychosocial disabilities’ may require reconfiguring the work environment and working hours. The participants explained that reasonable accommodation in the workplace needs to be supported, on the one hand, by better disclosure of individual needs and, on the other hand, by greater sensitisation of the employer.

The main additional out-of-pocket costs that were reported by participants related to travel to work. Many persons with more severe disabilities travel with an assistant, need specialised transport or make use of private transport. The use of an assistant is closely linked to the availability of accessible transport to places of work. Where accessible transport or subsidised, specialised services such as Dial-A-Ride or Sukuma were available, individuals experienced transport costs that were similar to the general population. In addition, participants reported cases of additional cost being incurred through inaccessibility of information about change in transport routes.

‘The problem is when the routes change. The driver asks all the passengers: if he can change his route on that day [*for whatever reason*] and the deaf person does not hear. Only later do you realize that the route has changed and then you have to get off and catch another taxi and it takes long and you pay double fares.’ (Person who is deaf, female, unemployed)

In order to enable meaningful participation in home, community and the workplace, we need innovations for universal design and reasonable accommodation, which target major cost drivers related to communication, housing, road infrastructure and transport.

## Discussion

Through using a participatory design, this article highlights the voices of persons with disabilities and their perception of disability-related out-of-pocket costs in South Africa. The study design has a number of limitations. Firstly, it was conducted in a very short period of time (three months), which allowed little time for data collection and restricted the researchers to interview persons only in three different areas of South Africa (Gauteng, Western Cape and KwaZulu-Natal) and those disability groups that were identified in the inception workshop. This allowed us to narrow down the investigation but also leaves out experiences of persons from other disability groups (e.g. albinism, dyslexia). Secondly, this article reports only on the qualitative part of the study and therefore only describes possible costs and is not representative for South Africa as a whole. Furthermore, current spending on disability-related costs for poorer socio economic groups is strongly curtailed by available income as well as limited knowledge about available goods and services. Hence, it is very likely that the cost of fulfilling actual needs is far greater than currently reported in this study.

The study reaffirms previous conclusions (Groce et al. 2011) that the link between disability and poverty in an MIC such as South Africa is more nuanced and complex than previously anticipated. It reiterates that persons with disabilities are a diverse group with households facing different costs related to care and support, accessibility of essential services and participation in home, community and workplace. The ‘borders’ between these needs are ‘blurred’ and interrelated which creates additional challenges for policy makers and programmes.

Similar to other studies (Banks & Polack 2013; Graham et al. 2013; Mall et al. 2014; Mitra 2006; Mitra et al. 2011; Palmer et al. 2015), this study identified costs that need to be addressed by a variety of role players (e.g. education, health, transport and social development). However, the results also suggest that planning to address economic vulnerability of persons with disabilities needs to also consider three different interlinked levels of care and support: (1) provision of care and support to enable survival and safety such as assistance, essential medical items and assistive devices, (2) provision of physically and financially accessible essential services (education and health) and (3) development of inclusive work and home environments that enable participation, in particular in income-generating activities.

Firstly, in MICs, interventions targeting survival (e.g. life-saving medication) are often prioritised before interventions that are perceived to improve quality of life only. Needs of disability (e.g. the provision of appropriate assistive devices and rehabilitation) are often seen as affecting quality of life and not survival. Yet, the voices of persons with disabilities highlight this as a misconception. As our examples highlighted, the absence of assistance and support for some conditions may be as life-threatening as the absence of medication (for conditions such as for TB or HIV). For instance, a quadriplegic person can die of pressure sore-related complications if they do not receive the necessary care and support. Similarly, a deaf-blind person without a translator is not able to communicate about his or her health and therefore cannot seek health care, which can lead to life-threatening situations. Hence, prioritising the needs of people with disabilities in national programmes is therefore a necessity to address economic vulnerability as well as to reinforce the right to live.

Secondly, the need to improve access to essential services fits within current developments of the South African National Health Insurance (NHI), which embraces the SDG idea of Universal Health Care access (Murnane et al. 2013). Approaches like the one proposed through ‘the ideal clinic’ concept need to better include elements of disability. For instance, the 2015 draft version of the South African Ideal Clinics manual includes a checklist speaking to the physical accessibility of clinics leaving out any other cost such as transport, sign language interpretation, linkages to rehabilitative services or provision of repair of assistive devices (South African Department of Health 2015). The 2016 version of the Ideal Clinics manual does not include this list and does not mention disability (South African Department of Health 2016).

This development is in disjunction with the introduction of the new Framework and Strategy on Disability and Rehabilitation Services (South African Department of Health 2015a), which was released at the end of 2015. This framework clearly identifies, amongst other things, the inaccessibility of health services and school settings as well as the need to improve and integrate disability and rehabilitation services. It is, however, not speaking about issues of some subgroups such as those with learning or intellectual disabilities (South African Department of Health 2015a). A review of the new developed policies with regard to their inclusion of disability services and accessibility seems to be urgently needed.

Hence, these two developments should be taken up as an opportunity to review how care and support, rehabilitation and assistive devices are provided, maintained and financed within the public health care system. It is an opportunity to review and re-engineer health care and related services in light of the CRPD concepts of ‘universal design’ and ‘reasonable accommodation’. This process needs to consider which products and services are needed for survival and access and whether these are included in the benefits package under the new NHI.

Thirdly, participation in society and, in particular, in income-generating activities is key to addressing economic vulnerability. In the context of high levels of unemployment, this may be neglected. However, recent research (Banks & Polack 2013; International Labour Office 2009) builds the argument that exclusion is costly to society at large and that exclusion reduces productivity and shifts costs to the person with disabilities and their households. Our study reinforces this argument. Hence, countries need to promote disability-inclusive work and home environments for their overall growth but also to economically protect households of persons with disability. This protection needs to be linked to individual care and support needs and includes a number of measures such as prioritisation in employment, accessible support services such as transport and housing.

In order to reduce economic vulnerability, South Africa has introduced a number of social protection mechanisms in the form of grants as well as a tax rebate system that accounts for disability-driven costs. The current grant system includes a disability grant and a care dependency grant. The disability grant currently includes two sets of eligibility criteria: the disability determination itself and an income threshold. A number of authors have reviewed the impact of the grant and identified that it reduces income poverty (but not to an equal level) for some groups but leaves out groups with less visible disabilities (Jelsma et al. 2008; Johannsmeier 2007; Mitra 2010). Authors have also described the use of the grant which covers costs of basic living for the whole family as well as costs specifically related to the person with disabilities (Booysen 2004; CASE 2005; De Koker et al. 2006; Macgregor 2006). This literature also highlights that the grant is often not used for disability-specific out-of-pocket costs (De Koker et al. 2006). Hence, some authors highlight the need to discuss the purpose of the grant. In other words, if the disability grant is thought to be more than a poverty grant, it needs to respond to the opportunity costs (income poverty) as well as the increased care and support costs (for survival, access and participation). Although previous studies particularly discuss who has (and has not) access to social protection mechanisms, such as the disability grant, and what the grant is being used for, our study suggests that the current ‘one size fits all’ approach (e.g. disability grant) does not respond to the diversity of needs and costs. This creates additional challenges for policy makers who need to design social protection mechanisms which are feasible to administer and which target diverse needs. How best to do this in an MIC is even internationally still a matter of discussion (International Labour Office & International Disability Alliance 2015). Much more research is needed to describe and identify the costs related to the diverse needs of care and support for the diverse group of persons with disabilities to inform how MICs can move towards addressing disability related-poverty in line with the golden standard laid out in the CRPD and new SDGs.
